# RETRACTION: Effects of Laparoscopic Radical Prostatectomy on Wound Infection of Surgery in Patients with Prostate Cancer: A Meta‐Analysis

**DOI:** 10.1111/iwj.70455

**Published:** 2025-03-26

## Abstract

**RETRACTION:** LiC. , WanZ.‐Q. , ZhengD.‐B. , and WangY.‐L. , “Effects of Laparoscopic Radical Prostatectomy on Wound Infection of Surgery in Patients with Prostate Cancer: A Meta‐Analysis,” International Wound Journal 21, no. 2 (2024): e14774, 10.1111/iwj.14774.38361180 PMC10869662

The above article, published online on 15 February 2024, in Wiley Online Library (http://onlinelibrary.wiley.com/), has been retracted by agreement between the journal Editor in Chief, Professor Keith Harding; and John Wiley & Sons Ltd. Following an investigation by the publisher, all parties have concluded that this article was accepted solely on the basis of a compromised peer review process. The editors have therefore decided to retract the article. The authors did not respond to our notice regarding the retraction.

**RETRACTION:** C. Li , Z.‐Q. Wan , D.‐B. Zheng , and Y.‐L. Wang , “Effects of Laparoscopic Radical Prostatectomy on Wound Infection of Surgery in Patients with Prostate Cancer: A Meta‐Analysis,” International Wound Journal 21, no. 2 (2024): e14774, 10.1111/iwj.14774.38361180 PMC10869662

The above article, published online on 15 February 2024, in Wiley Online Library (http://onlinelibrary.wiley.com/), has been retracted by agreement between the journal Editor in Chief, Professor Keith Harding; and John Wiley & Sons Ltd. Following an investigation by the publisher, all parties have concluded that this article was accepted solely on the basis of a compromised peer review process. The editors have therefore decided to retract the article. The authors did not respond to our notice regarding the retraction.

